# Low-intensity pulsed ultrasound triggers a beneficial neuromodulation in dementia mice with chronic cerebral hypoperfusion via activation of hippocampal Fndc5/irisin signaling

**DOI:** 10.1186/s12967-022-03824-7

**Published:** 2023-02-23

**Authors:** Degang Song, Xiaomin Chen, Na Zhou, Yi Yuan, Shuo Geng, Cong Zhang, Zhe Zhao, Xingran Wang, Xinran Bao, Xifa Lan, Xiangjian Zhang

**Affiliations:** 1 Department of Neurology, First Hospital of Qinhuangdao, Hebei Medical University, No. 258, Wenhua Road, Qinhuangdao, 066000 Hebei China; 2 Hebei Key Laboratory of Vascular Homeostasis and Hebei Collaborative Innovation Center for Cardio-cerebrovascular Disease, No. 215, Hepingxi Road, Shijiazhuang, 050000 Hebei China; 3 Department of Nursing, First Hospital of Qinhuangdao, Hebei Medical University, No. 258, Wenhua Road, Qinhuangdao, 066000 Hebei China; 4 Institute of Electrical Engineering, Yanshan University, No. 438, Hebei Street, Qinhuangdao, 066000 Hebei China; 5 Department of Biological Sciences, Virginia Tech, Blacksburg, VG 24061 USA; 6 Department of Neurology, Second Hospital of Hebei Medical University, No. 215, Hepingxi Road, Shijiazhuang, 050000 Hebei China

**Keywords:** Fndc5/irisin, LIPUS, Dementia, Hippocampus, Chronic cerebral hypoperfusion, Neurons, Astrocytes

## Abstract

**Background:**

Exercise-related signaling Fndc5/irisin expresses in brain and acts as a crucial regulator of cognitive function, but its detailed roles in vascular dementia (VaD) are still unclear. Low intensity pulsed ultrasound (LIPUS), a novel brain stimulation approach, has been suggested as a promising treatment for dementia. Here, we investigated the activity and efficacy of Fndc5/irisin in experimental VaD, further explored whether the potential effects of LIPUS on VaD is related to Fndc5/irisin.

**Methods:**

Mouse model of VaD was established with chronic cerebral hypoperfusion (CCH) using bilateral common carotid arteries stenosis (BCAS). Transcranial LIPUS was applied 24 h after BCAS and subsequently daily with a stimulation time of 5 min at an ultrasound pressure of 0.51 MPa for a period of 28 days. The levels of Fndc5/irisin in different brain regions, the hippocampal long-term potentiation and anti-inflammatory cytokines were investigated at day 28 after cognitive evaluation. Global Fndc5 knock-out (F5KO), forced expression or knockdown of Fndc5, and recombinant irisin application were respectively employed for mechanism exploration. The neuron dendritic spine density and astrocyte phenotype were detected in vitro.

**Results:**

Fndc5/irisin was reduced in hippocampus of BCAS mice, forced expression hippocampal Fndc5 or bilateral intrahippocampal injection of recombinant irisin respectively improved hippocampal synaptic plasticity or inflammatory microenvironment, and then alleviated the cognitive impairments. LIPUS existed a positive efficacy in enhancing hippocampal Fndc5/irisin in BCAS mice, thus triggering a beneficial neuromodulation for VaD protection. Importantly, the neurorestorative effects of LIPUS on CCH-induced damages were totally reversed by knockdown the expression of hippocampal Fndc5 in WT mice, or in F5KO mice. Moreover, Fndc5 mediated the upregulated effects of LIPUS on spine density as well as irisin secretion of hippocampal neurons. The neuron-secreted irisin further drove reactive astrocytes to a neuroprotective phenotype.

**Conclusion:**

LIPUS induced a neurorestorative stimulation against VaD may be through upregulation of the hippocampal Fndc5/irisin levels. Hippocampal Fndc5/irisin signaling might be a promising strategic target for VaD.

## Introduction

Chronic cerebral hypoperfusion (CCH) is the basic pathophysiological characteristic of vascular dementia (VaD), and contributes to long-term cognitive deficits and memory loss through complex molecular and pathway mechanisms [1, 2]. Hippocampus, a crucial brain region centrally involved in memory and learning is susceptible to injured by CCH, resulting in the local destroyed synaptic plasticity and dysregulated neuroinflammation [3, 4]. Although marked progress has been achieved over the past decades, unlike stroke and Alzheimer’s disease (AD), there are still no licensed treatment of VaD [5, 6]. Therefore, identifying the critical mechanism regulating the development and progression of VaD, and adopting targeted treatments is a major public health problem to be solved urgently.

Exercise, especially endurance exercise, is well known to have multiple benefits on body healthy, such as optimizing lipid metabolism, promoting bone growth and improving cognitive function [7, 8]. Fibronectin-domain III containing 5 (Fndc5) is a glycosylated type 1 membrane protein mainly expressed by muscle fibers, and has been identified as a central exercise-regulated factor that mediates major metabolic benefits [9, 10]. Irisin is a newly identified exercise hormone released from the cleavage of Fndc5, which mediates its effects on bone and fat via integrin-αVβ5 receptors [9, 11]. Recently, the detection of Fndc5 in hippocampal neurons and irisin in the cerebrospinal fluid (CSF) opened a new chapter for neuroscience study [12]. Considerable evidences highlighted that Fndc5/irisin mediates the expression of brain-derived neurotrophic factor (BDNF) in hippocampal neurons [13], and has neuroprotective effects against neurodegenerative diseases and neuroinflammation [14]. Fndc5/irisin has been found to be reduced in AD brain, and forced expression of Fndc5 in the brain or bilateral intrahippocampal injection of recombinant irisin can rescue hippocampal synaptic plasticity as well as cognitive dysfunction in mouse AD models [15]. Thus, hippocampal Fndc5/irisin might be a critical molecular mechanism of dementia and potential to become a novel therapeutic target for dementia.

Ultrasound therapy has an exciting and compelling history of medicine applications. Low-intensity pulsed ultrasound (LIPUS) is a specialized form of ultrasound that delivers low-intensity outputs in a mode of pulsed waves, and able to transmit acoustic energy to target tissue without thermal effects [16]. Currently, transcranial LIPUS has been considered as the only neuromodulation modality that combines noninvasiveness and depth penetration [17], which gets more attention in exploration of novel treatment for dementia [18, 19], and some related studies are already in the preclinical stage [20, 21]. Growing evidences demonstrate the multiple neuroprotective effects of LIPUS on neuroinflammation and neurodegenerative diseases [22–24], which appears to be similar to the effects of Fndc5/irisin. For instance, both LIPUS and Fndc5/irisin signaling can precisely mediate the expression of BDNF in hippocampal neurons [13, 19]. As a widely accepted noninvasive technology for neuromodulation, LIPUS is highly expected to be a promising therapy for dementia, however, the exact mechanism of the LIPUS action is largely unclear, which may hinder its accurate application [25].

Recently, studies involving the Fndc5/irisin signaling are booming in the dementia field [26], however, the role of Fndc5/irisin in VaD has not been investigated. Here, we investigated the Fndc5/irisin in mouse injured by CCH, and explored whether it could be a critical mechanism for therapeutic intervention in experimental VaD. Moreover, the similar neuroprotective effects of Fndc5/irisin and LIPUS led us to further tested the hypothesis that LIPUS protects VaD via modulating the activity of Fndc5/irisin signaling. Here we show that hippocampal Fndc5/irisin is reduced in mice with CCH, and boosting hippocampal Fndc5/irisin levels rescue hippocampal damage and memory defects. Furthermore, our data indicated that transcranial LIPUS could be a qualified non-invasive technology to improve the impaired Fndc5/irisin signaling in hippocampus, and then treat the brain disorders related to CCH.

## Materials and methods

### Animals

Male WT mice (C57BL/6, weight 28.0–32.0 g, 2 or 9 months of age) of specific pathogen-free grade were obtained from the Vital River Laboratory Animal Technology (Beijing, China). FNDC5^−/−^ mice (F5KO) on a C57BL/6 background were purchased from BioMedical Research Institute of Nanjing University (Nanjing, China). The animals were accommodated in cages in a room with a 12 h light-dark cycle, with constant temperature at 25 ± 1 °C, and with a humidity at 55 ± 5%. The experimental animals were housed in groups of five per cage with ad libitum access to standard laboratory diet and water. The animal experiments in this study were performed according to the international ethical statutes and law for the protection of animals. All procedures involving animal experiment and gene editing were approved by the Medical Ethics Committee of the Hebei Medical University (HMUSHC-130318).

### BCAS procedure

Mouse VaD model was established using bilateral common carotid artery stenosis (BCAS) as previously described [27]. Briefly, Mice were deeply anesthetized using 2.5% isoflurane (Cristália; São Paulo, Brazil) by using a vaporizer system (Norwell, MA). Anterior neck skin was disinfected with 75% alcohol before making a midline cervical incision. the right common carotid artery was gently exposed, the artery was wrapped with a micro-coil (0.18-mm internal diameter). After 1 h, the same procedure was performed on the left common carotid artery. The rectal temperature of mice was maintained at 37 ± 0.5 ℃ during surgery using a heated blanket (Harvard Homoeothermic Blanket Control Unit, Harvard Apparatus, MA, USA). A penicillin sodium solution was covered on the wounds to prevent infection. Carprofen (5 mg/kg, i.p., Vet One) were administered on the day of surgery and daily for the next 72 h to alleviate pain, and attenuate inflammation. Sham-operated mice were subjected to the same surgery, but not wrap the common carotid artery. Regional cerebral blood flow (rCBF) of bil. hippocampus was monitored after BCAS immediately and 14 days, The mice without definite decline of hippocampal rCBF were excluded in further experiments.

### Brain infusions

Forced expression of Fndc5 in adult C57BL/6 mice brain were infected via intracerebroventricular injection (coordinates relative to Bregma: 0.2 mm AP;1.0 ML; 2.4 mm DV) with an adenoviral vector designed to express Fndc5 (AdFndc5) as previously described [15], vectors designed to express GFP (AdGFP) was used as control. AdFndc5 or AdGFP produced by Viraquest Inc. (North Liberty, IA) using the Gateway expression system (Life Technologies) were injected into right ventricle (1.0 × 10^8^, per animal). Behavioral and electrophysiological studies were carried out 6 days post-injections. Knockdown the expression of Fndc5 in hippocampi was achieved by bilateral intrahippocampal injection (coordinates relative to Bregma: 2.0 mm AP, ± 1.5 mm mL, 1.5 mm DV) of lentiviral particles expressing shRNA against murine Fndc5 (shFndc5) as previously described [13, 15], an innocuous shRNA targeting luciferase (shLuc) used as control. shFndc5 or shLuc (titer of 1.0–2.0 × 10^9^ particles/ml, Sigma-Aldrich, St Louis, MO) were stereotaxically injected into bilateral hippocampus region (1 µl, per animal). Recombinant irisin (75 pmol per site, Phoenix Pharmaceuticals, Burlingame, CA) was bilaterally delivered into the hippocampal region (stereotaxical coordinates relative to Bregma: 2.0 mm AP, ± 1.5 mm mL, 1.5 mm DV) [15]. Before those brain infusions, mice were anesthetized with 2.5% isoflurane and then carefully affixed to a stereotaxic frame. Mice that showed signs of misplaced injections or any sign of hemorrhage were excluded from further analysis.

### Pulsed ultrasound in mice

LIPUS used in in vivo experiments was generated by a 1-MHz single-element focused transducer (A392S; Panametrics, Waltham, MA, USA) with 50 ms burst lengths at a 5% duty cycle and a repetition frequency of 1 Hz. a 3D-print coupling cone was used to connect the mouse skull and the transducer. The sonication was precisely targeted using a stereotaxic apparatus (Stoelting, Wood Dale, IL, USA). The acoustic wave was delivered from the top of the mouse brain to the desired region containing bilateral hippocampus. The irradiation range of LIPUS cover bilateral hippocampus and part of forebrain. LIPUS was applied 24 h after BCAS and subsequently daily for a period of 28 days with a sonication time of 5 min at an acoustic power of 0.51 W (corresponding to a spatial-peak temporal-average intensity (I_SPTA_) of 528 mW/cm^2^), the intensity and scheme of the LIPUS exposures were selected according the previous studies [28]. A function generator (33220 A, Agilent Inc., Palo Alto, USA) was connected to a power amplifier (500-009, Advanced Surgical Systems, Tucson, AZ) to create the Ultrasound excitation signal. A power meter/sensor module (Bird 4421, Ohio, USA) was used to measure the input electrical power. The heads of mice were fixed during LIPUS procedure. Mice that showed any sign of hemorrhage or new-onset neurological symptoms were excluded from further analysis.

### Behavioral testing

The novel object recognition task relies on the tendency of exploring novelty. It is used for assessing non-spatial object memory in rodents [29]. In short, mice were set in 30 × 30 × 45 cm open field arena for 5 min, in which they could explore the empty arena freely. After this habituation session mice were trained in a 5 min-long session, two identical objects for exploring were placed on the front of each animal, which was placed at the center of the arena. The amounts of time to explore each object was recorded by trained researchers. 1 h after training, mice were re-exposed into the test session arena, with one of the previously explored objects replaced by a novel object. Exploratory time of the familiar and the novel objects were gauged. Mice that had amounts of time below 10 s were excluded from this test. The open field arena and each object were wiped thoroughly with 70% ethanol between behavioral procedures in order to eliminate olfactory cues. Results were expressed as percentage of time exploring each object during the training or test session, then, these data were analyzed using a one-sample Student’s t test comparing the mean exploration time for each object with the fixed value of 50%.

Contextual fear learning is used to assess memory and is based on the tendency of mice to show a fear response (freezing) when re-exposed to the context where they received an aversive stimulus (in this case, foot shock). A time-sampling procedure was used to observe freezing behavior at training and testing time [29]. Each mouse was allowed to explore the conditioning chamber (40 × 25 × 30 cm) freely for 2 min, and then, a two-sec shock stimulus (0.8 mA) was called to the floor. Mice remained in the chambers for an addition min before being returned to their home cages in the holding room. Approximately 24 h later, mice were placed in the same chamber over 5 min without electric shock. Freezing behavior was recorded during the period, then the percentage of time in freezing events was calculated by the Ethovision software (Noldus, Leesburg, VA) for each mouse.

The radial arm water maze is characterized by the spatial complexity and simplicity of performance measurement without requiring foot shock or food deprivation as motivating factors, which was constructed as described previously [30]. The equipment consists of a diameter of 20 cm open central area (a circular black water pool) radiating out six swim arms (30 cm in length, 8 cm in width), the pool was filled with water (the pool temperature should be 20 ℃ − 22℃ ) to a depth of 16 cm, with an escape platform located in a defined arm of the device. The goal arm is held constant for all trials. The visual cues consisted of black and white posters placed on the surrounding walls. mice were examined for their ability to find the escape platform along 30 trials in two days. At day 1, 5 training blocks were implemented with alternation between visible and hidden platform. On day 2, all 5 blocks were performed with hidden platform. A block comprises 3 trials of 60 s each. The number of entries into incorrect maze arms (errors) was averaged per block. After each trial, the mouse was dried by gently towel and warm up by 150-W floodlight for 30 min before placing them back into home cages.

### Tissue acquisition

After the behavioral testing test, Mice were anesthetized with 2.5% isoflurane and euthanized using cervical dislocation. The serum, CSF, forebrain, cerebellum and hippocampus were instantly collected according the previous studies [13, 15, 29].

### Hippocampal long-term potentiation (LTP) measurements

Transverse hippocampal slices (400 μm) were harvested using a cryostat microtome (LEICA CM1850, Leica Biosystems, Wetzlar, Germany), and placed on infusion chambers with aCSF. Field excitatory postsynaptic potentials (fEPSPs) were recorded from hippocampal region according to previously established procedures [31]. Slices were permitted to recover for at least 90 min before recording. Signals from the amplifier (Axoclamp-2 A) were recorded with the thermal pen recorder (DC-1, Nihon Kohden, Tokyo, Japan). The data were stored and subsequently analyzed using pCLAMP 10 (Axon Instruments) data acquisition and analysis program on a personal computer. The electrophysiological recordings were performed at 36.5 ± 0.5 ℃.

### Western blotting

The protein level of Fndc5/irisin in hippocampus were detected using Western blotting. The detailed proposal was according our previous study [32]. The primary antibody: anti-Fndc5/irisin (1:1000, Abcam, #ab131390), anti-β-actin (1:20,000, Abcam, #ab8226).

### Primary cell culture

Primary hippocampal neuron cultures were prepared using fetal C57BL/6J (embryonic 18–20 days) according to previous protocol [33]. Embryos’ heads were rapidly decapitated and placed in Hank’s balanced salt solution (HBSS) buffer (Gibco). The whole brains were removed carefully and placed in fresh HBSS buffer immediately. Ventrally, hippocampi were taken out from midbrain where was between the diencephalon and striatum to the caudal hippocampi (approaching base of the brain). And then, hippocampi were blunt dissected from the corpus callosum and cortex, and transferred to a 15 ml conical tube containing 9 ml HBSS. When hippocampi were deposited to the bottom of tubes, 4.5 ml of HBSS was replaced by 0.5 ml 2.5% trypsin (final concentration of 0.25%) and 50 µl of DNAsel (final concentration of 1 mg/ml). The hippocampi were digested in chamber at 37℃ for 15 min. The digestion was terminated by plating medium (Neurobasal medium supplemented with 10% FBS, 1% GlutaMAX, 1% sodium pyruvate, and 1% penicillin-streptomycin). Hippocampi were dissociated and then filtered by a 40 µm cell strainer. The resulting cell suspension was filtered by another 40 µm cell strainer, and 1 × 10^6^ cells were seeded with plating medium on coverslips pre-coated with Poly-D-Lysine (PDL, Sigma) in 24-well plate. The plating medium was replaced with culture medium (Neurobasal medium supplemented with 1% GlutaMAX, 1% penicillin-streptomycin, and B-27 Plus supplement, Gibco) after 4 hours. Half and one-third of the medium were removed with culture medium containing 10 µm 5-fluoro-2’-deoxyuridine (FdU, Gibco) refreshed every 2–3 days.

Hippocampal primary astrocytes cultures were obtained and purified from C57BL/6J mouse pups within 24 h after birth as previously documented [34]. In brief, in detail, the meninges of pups’ hippocampi were removed and then digested with papain (Worthington Biochemical Corporation). The mixture was filtered through 70 μm cell strainer and seeded into 75 cm^2^ flasks coated by poly-L-lysine for generating mixed-glia. The non-adherent cells were removed by medium changed after 24 h. Cultures were maintained in DMEM media with low glucose supplemented with l-glutamine, 10% FBS and 1% penicillin-streptomycin. Medium was refreshed every 3–4 days until 12 days. The astrocytes were isolated from mixed cells by shaking flasks at 37 ℃ at 300 rpm for 4–6 h in an orbital shaker. Approximately 95% of the isolated cells were confirmed as astrocytes by glial fibrillary acidic protein (GFAP) staining.

### Oxygen and glucose deprivation

Oxygen and glucose deprivation (OGD) was employed to mimic VaD ischemic damage in vitro as previously described [27]. Primary astrocytes or neurons were cultured in the corresponding medium without serum and glucose, and incubated for 6 h in a humidified incubator containing 5% CO_2_ and 95% N2 at 37 °C.

### **Pulsed ultrasound** in vitro

Pulsed ultrasound was performed in cell cultures as previously described [35]. A 1-MHz plane piezoelectric transducer (A394S-SU, Panametrics, Waltham, MA, USA) was used to generate LIPUS with 50 ms burst lengths at a 50% duty cycle and a repetition frequency of 10 Hz. The acoustic wave was transmitted from the plane transducer to the bottom of the cell culture plate and daily applied for a stimulation time of 5 min with a I_SPTA_ of 110 mW/cm^2^ at a period of 2 days. Ultrasound transmission gel (Pharmaceutical Innovations, Newark, NJ, USA) was employed to fill the area between the transducer and the plate or the brain to maximize the ultrasound transmission.

### **Cell infusions**

Three different lentiviral vectors targeting Fndc5 (shFndc5-1, shFndc5-2 and shFndc5-3) were obtain from Sigma-Aldrich (St Louis, MO), and used for knockdown the Fndc5 in primary hippocampal neurons [13]. The shFndc5 (10^6^/mL) were directly added to cell culture medium and allowed to express for five days before cells were processed for other detection.

### Cell viability evaluation

Cell viability of cultured neurons and astrocytes were assessed using Cell Counting Kit 8 (CCK8, Kumamoto Dojindo, Japan) according to the manufacturer’s instructions.

### Immunohistochemistry

Cells were seeded in coverslips and fixed in ice-cold 4% formaldehyde plus 4% sucrose for 10 min, and accepted 0.1% Triton permeabilization and serum albumin blocking. Astrocytes were incubated overnight at 4 °C with the following primary antibodies: rat anti-GFAP (1:500, Abcam), rabbit anti-S100A10 (1:200, Abcam) and mouse anti-Integrin αVβ5 (1:100, Millipore MAB1961). The next day, coverslips were further incubated with corresponding secondary antibody (Alexa Fluor 488 or 594, Jackson Immuno Research, USA) for 1 h. Neurons were incubated overnight at 4 °C with Alexa-conjugated Phalloidin (2 U per coverslip, Sigma-Aldrich). Nuclei were counterstained in DAPI containing Prolong mounting solution. Coverslips were imaged on a Zeiss AxioObserver Z1 microscope. 20–30 images (from 2 to 3 coverslips) were acquired per experimental condition for each experiment (three experiments with independent neuronal cultures).

Determination of the percentage of double positive cells was achieved by counting the S100A10^+^ and GFAP^+^ co-labeled cells using the ImageJ software [32]. The percentage of co-labeled cells was calculated as: (100% × Total number of double positive cells/Total number of GFAP^+^ cells.

Spine density was assessed as described previously [15], two or three distal dendrite segments were isolated per neuron, number of spines was manually determined by a researcher blind to experimental conditions. Results are expressed as the mean number of spines per µm.

### ELISA

The concentration of irisin in serum, brain tissue homogenate or culture supernatant were detected using the irisin ELISA kit obtained from Phoenix pharmaceuticals (Burlingame, USA). and were obtained from Phoenix pharmaceuticals (Burlingame, USA). transforming growth factor (TGF)-β, and interleukin (IL)-10 were measured with mouse TGF-β and IL-10 ELISA Kits (Aviscera Bioscience, Santa Clara, CA, USA). After optimized sample dilution, ELISA were performed according to the manufacturer’s instructions.

### Real-Time PCR

Total mRNA was harvested from brain tissue or hippocampal primary cells using TRIZOL reagent (Invitrogen, Carlsbad, CA, USA) according to the manufacturer’s instructions. cDNA was obtained using a Rapid Reverse Transcription Kit (Cat No.: 11752-250, Invitrogen). The sequences of the specific primers for target genes are listed in (Invitrogen, Table 1).

**Table 1:** Primers used for qRT–PCR

Primers	Forward (5′–3′)	Reverse (5′–3′)
Fndc5	CATGTTTCCTTAGCTCTACTGTG	GGAGAAAGCATGCATGGCAGTCT
S100A10	AAAGACCCTCTGGCTGTGG	AATCCTTCTATGGGGGAAGC
Integrin-αV	GTTGGGAGATTAGACAGAGGA	CAAAACAGCCAGTAGCAACAA
Integrin-β5	ACTATCCATCCCTTGCCTTGCTTG	GCAGTCCTTGGCGGT TTTGTAGAA
GAPDH	GGAGCGAGATCCCTCCAAAAT	GGCTGTTGTCATACTTCTCATGG

### Statistical analysis

A total of 259 male mice were used in this present study, of which 190 were adult WT mice, 42 were aging WT mice and 25 were F5KO mice. 3 aging WT mice as well as 2 F5KO mice with unqualified behavioral training, and 12 adult WT mice that showed signs of misplaced injections or any sign of hemorrhage during brain infusion were excluded from further analysis. Finally, 242 mice were used for data analysis and randomly assigned to different experimental groups: Sham + BCAS group (aging, n = 16; adult, n = 16), Sham + BCAS + AdFndc5 group (adult, n = 24), Sham + BCAS + Irisin group (adult, n = 24), Sham + BCAS + LIPUS group (aging, n = 24; adult, n = 24; F5KO, n = 24), Sham + BCAS + LIPUS + shLuc + shFndc5 (adult, n = 90). Sample sizes were based on previous published study [15] and our unpublished work using LIPUS, as well as calculations from pilot data to determine the minimum number of animals needed to achieve a power level of 80% for key measures.

All outcome analysis were carried out by independent researchers blinded to the information of experimental groups. GraphPad Prism 8.0 was used to perform all statistical analysis. Data were presented as mean ± SEM, unless otherwise stated. One-way ANOVA followed by Tukey’s post hoc test was used to test the differences among multiple groups. Repeated measures two-way ANOVA was used to compare the time-course data including the LTP measurement and the errors evaluation. Student’s t-test was used to test the differences among two groups. One-sample t-test was used to compare the data of novel object recognition task. Differences were considered significant at *P* < 0.05.

## Result

### Fndc5/irisin is reduced in hippocampus of BCAS mice

**Fig. 1:**
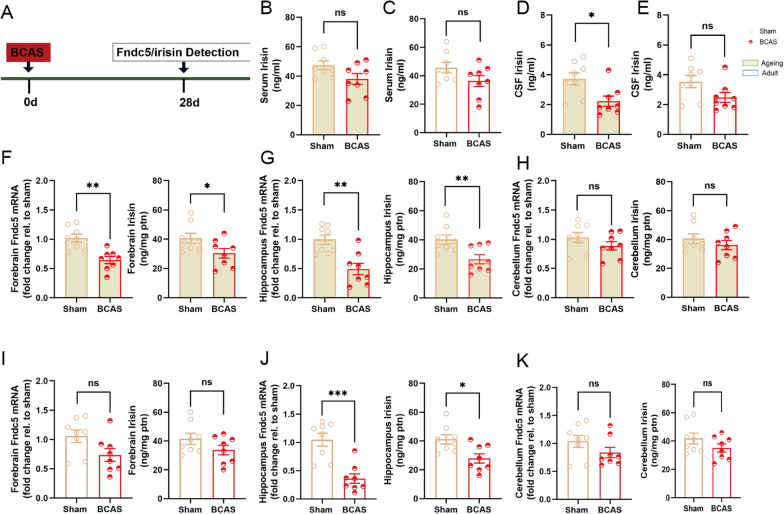
Hippocampal Fndc5/irisin is reduced in mouse VaD models. **A** Experimental outline. Experimental vascular dementia was established in C57B/6 mice using BCAS. Fndc5 mRNA expression or irisin concentration were respectively assessed using qPCR or ELISA on day 28 after BCAS. **B** Serum irisin of ageing mice. **C** Serum irisin of adult mice. **D** CSF irisin of ageing mice. **E** CSF irisin of adult mice. **F** Fndc5 expression and irisin in forebrain of ageing mice. **G** Fndc5 expression and irisin in hippocampus of ageing mice. **H** Fndc5 expression and irisin in cerebellum of ageing mice. **I** Fndc5 expression and irisin in forebrain of adult mice. **J** Fndc5 expression and irisin in hippocampus of adult mice. **K** Fndc5 expression and irisin in cerebellum of adult mice. Sham, sham-operated mice; BCAS, mice were subjected to Bilateral common carotid arteries stenosis. (n = 8 per group. All data are expressed as mean ± SEM, *P < 0.05, **P < 0.01, ***P < 0.001, ns means no statistical significance)

Fndc5/irisin system exists in the brain, and has been known as a critical regulator of cognitive function [29]. Serum irisin has been found as a potential biomarker for VaD induced cognitive decline [36], but the state of Fndc5/irisin in VaD is not clear yet. In current study, Fndc5/irisin was detected in experimental VaD. Bilateral common carotid arteries stenosis (BCAS) was employed to establish VaD model in ageing (9 months of age) mice and adult (2 months of age) mice. Compared to the sham group, serum irisin had no significant change in ageing (*P* = 0.069; Fig. 1B) and adult (*P* = 0.099; Fig. 1C) BCAS mice, CSF irisin was deceased in ageing BCAS mice (*P* = 0.015; Fig. 1D) but not in adult BCAS mice (*P* = 0.060; Fig. 1E). BCAS injure induced significant reduction of Fndc5 gene expression as well as irisin concentration in forebrain (Fndc5 mRNA: *P* = 0.001, irisin: *P* = 0.049; Fig. 1F), hippocampus (Fndc5 mRNA: *P* = 0.001, irisin: *P* = 0.009; Fig. 1G) and cerebellum (Fndc5 mRNA: *P* = 0.220, irisin: *P* = 0.361; Fig. 1H) of ageing mice, suggesting the activity of Fndc5/irisin is totally inhibited in brain with CCH. Interestingly, Fndc5 gene expression as well as irisin level were only reduced in hippocampus (Fndc5 mRNA: *P* < 0.001, irisin: *P* = 0.012; Fig. 1J), while not in other brain regions of adult BCAS mice (Forebrain: *P* > 0.05, Fig. 1I, cerebellum: *P* > 0.05; Fig. 1K). Hippocampus is the most vulnerable region in brain suffering CCH and closely related to cognitive impairment. Our data confirmed hippocampal Fndc5/irisin is reduced in both ageing and adult mouse model of VaD, indicating the activity of hippocampal Fndc5/irisin may be damaged preferentially in VaD.

### Boosting hippocampal Fndc5/irisin alleviates BCAS-induced hippocampal damage and cognitive deficits

**Fig. 2:**
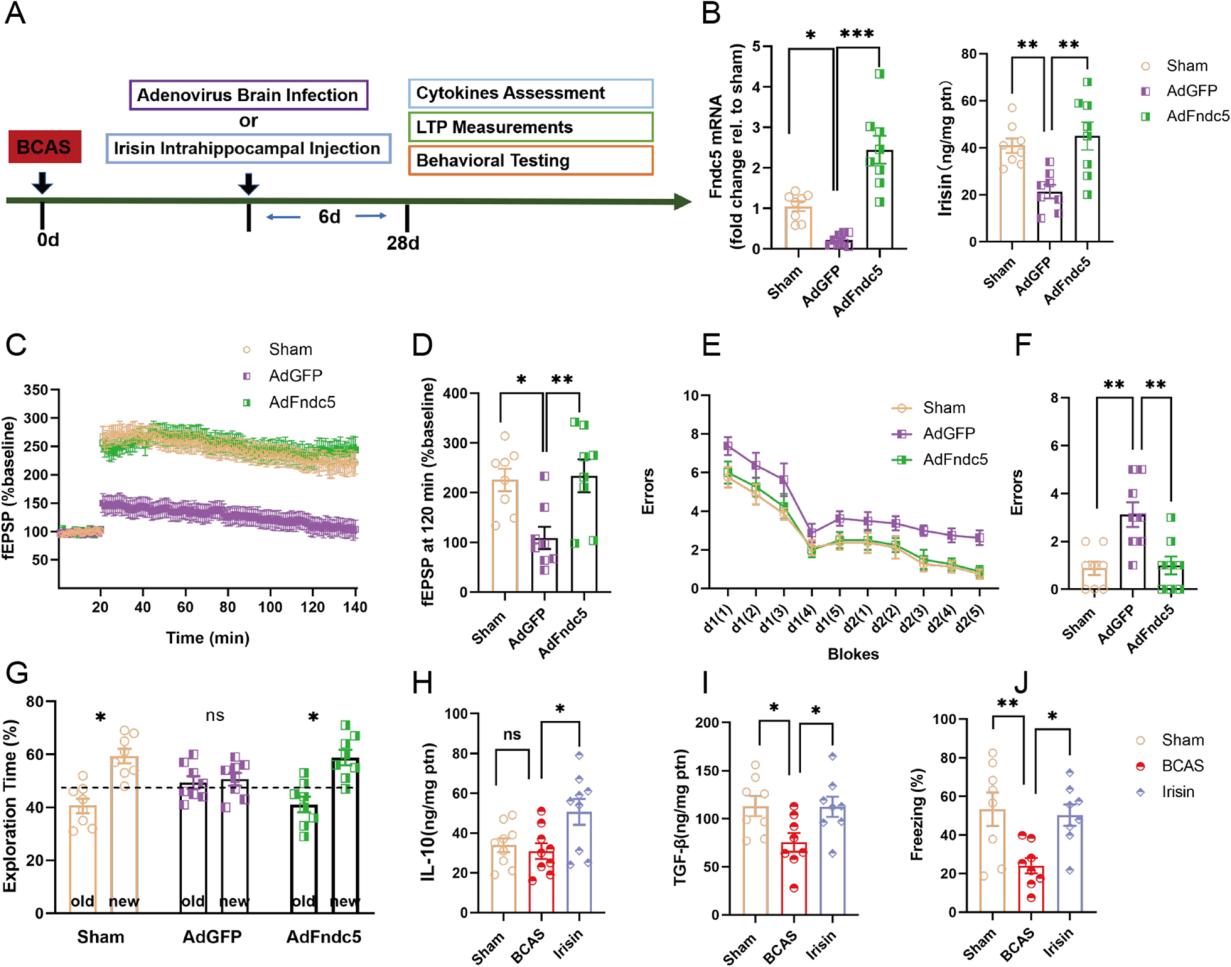
Boosting hippocampal Fndc5/irisin alleviate hippocampal damage and cognitive deficits following experimental VaD. **A** Experimental outline. Forced expression of Fndc5 in hippocampus of adult C57BL/6 mice using adenovirus brain infection, while enhancement of hippocampal irisin level by bilateral intrahippocampal injection of recombinant irisin on day 22 after BCAS. **B** Hippocampal Fndc5 mRNA expression and irisin were respectively detected using qPCR and ELISA on day 28 after BCAS. **C** Hippocampal synaptic plasticity was analyzed using Patch Clamp on day 28 after BCAS. The average traces of fEPSP in hippocampal slices. **D** The data of fEPSP at 120 min. **E**–**G** Cognitive evaluation were performed on day 28 after BCAS. The errors (numbers of entries into incorrect maze arms) in two-days radial arm water maze test were continuously measured in each block (**E**). The errors in last block were counted in a histogram (**F**). Exploratory time on old or new object in novel object recognition test were illustrated in histogram (**G**). **H**, **I** Hippocampal level of TGF-β and IL-10, were determined using ELISA on day 28 after BCAS. **J** The percentage of freezing time obtained on day 28 after BCAS in contextual fear test were illustrated in histogram. Sham, sham-operated mice; BCAS, mice were subjected to BCAS injury; AdFndc5 or AdGFP, adult BCAS mice were infected using intracerebroventricular injection with an adenoviral vector designed to express Fndc5 or GFP; Irisin, adult BCAS mice with bilateral intrahippocampal injection of recombinant irisin. (n = 8 per group. All data are expressed as mean ± SEM, *P < 0.05, **P < 0.01, ***P < 0.001, ns means no statistical significance)

Sustained neuroinflammation and destroyed synaptic plasticity are the characteristic damage in dementia hippocampus [37]. Uncontrolled hippocampal damage can cause serious cognitive deficits [3]. Previous studies have highlighted the protective effects of Fndc5/irisin on synaptic plasticity [38], neuroinflammation [14] and cognitive function [39]. We further investigated the potential roles of hippocampal Fndc5/irisin in experimental VaD. Although VaD always develops in old age, adult mice are widely used for neuroscience research and easy to obtain in sufficient quantities for molecular studies, therefore we choose the adult VaD model to explore the Fndc5/irisin-related mechanism. In present study, AdFndc5 brain infection increases hippocampal Fndc5 mRNA expression (*P* < 0.001, Fig. 2B) and irisin (*P* = 0.002, Fig. 2B) concentration in adult BCAS C57BL/6 mice compared to AdGFP brain infection. Interestingly, boosting Fndc5 expression in hippocampus strengthened the fEPSPs of Ex vivo hippocampal slices (AdGFP vs. AdFndc5: *P* = 0.008; Fig. 2C, D). Expectedly, AdFndc5 brain infection significantly decreased the errors of BCAS mice in two-day radial arm water maze test task compared to AdGFP infection (AdGFP vs. AdFndc5: *P* = 0.004; Fig. 2E, F). In novel object recognition task, AdFndc5 mice performed better and devoted more exploration time to the new subject than the AdGFP mice (AdFndc5: *P* = 0.001, AdGFP: *P* = 0.715; Fig. 2G). In addition to forced expression of Fndc5, we attempted to directly save the reduced hippocampal irisin using bilateral intrahippocampal injection of recombinant irisin, which improved hippocampal inflammatory microenvironment of BCAS mice by increasing the level of TGF-β (*P* = 0.046; Fig. 2I) and IL-10 (*P* = 0.021; Fig. 2H), as well as the cognitive deficits by enhancing the percentage of freezing time in contextual fear test (*P* = 0.021; Fig. 2J). In summary, boosting hippocampal Fndc5/irisin ameliorated the hippocampal inflammatory microenvironment as well as the destroyed hippocampal synaptic plasticity, and the cognitive deficits in mice with experimental VaD. Our data suggested hippocampal Fndc5/irisin signaling might be a critical mechanism determining the pathological process of VaD.

### LIPUS drives a bolstering of Fndc5/irisin in hippocampus of BCAS mice

**Fig. 3:**
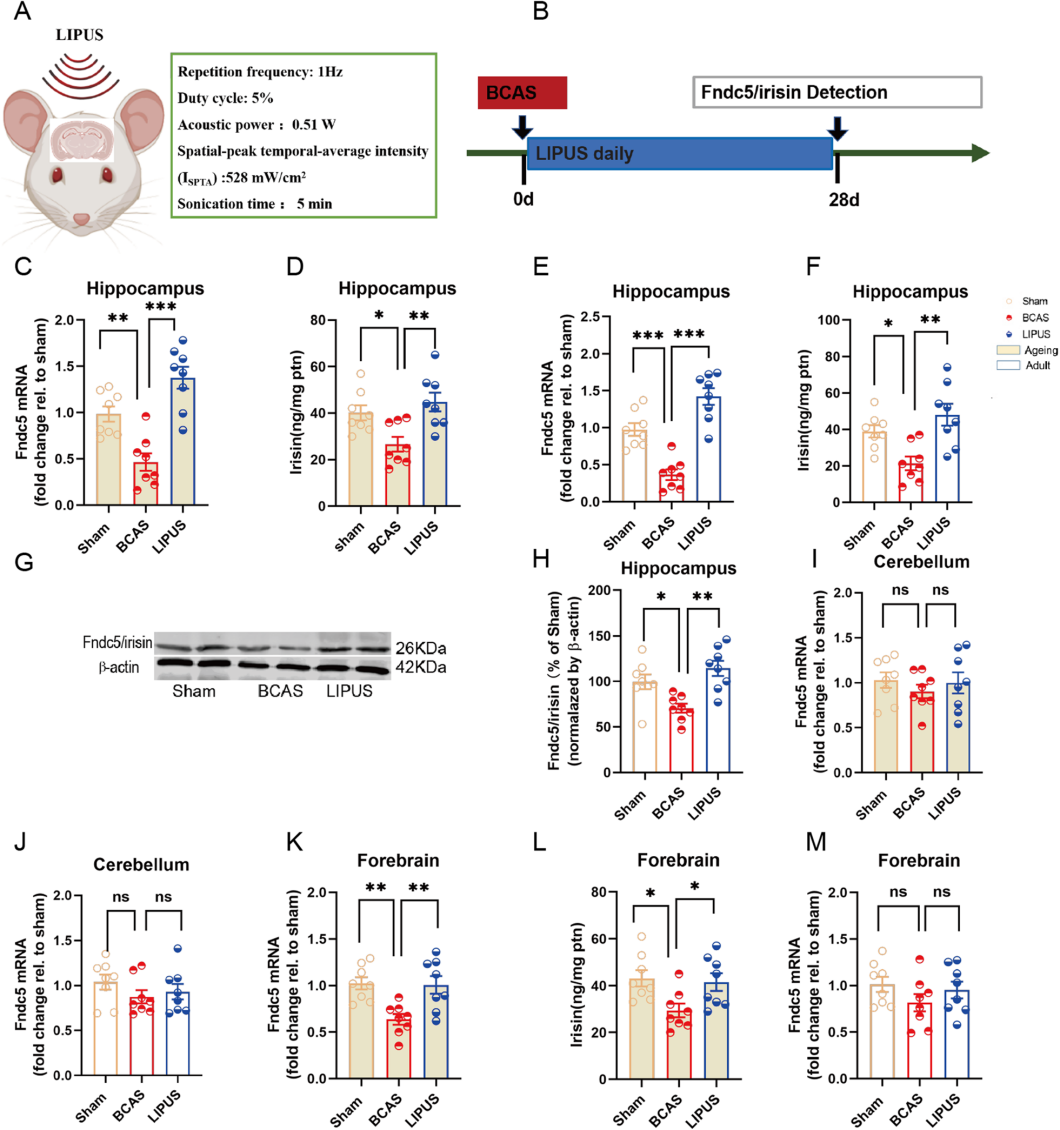
LIPUS induces a bolstering of hippocampal Fndc5/irisin in VaD mice. **A** Schematic diagram of LIPUS. The acoustic wave was delivered to the targeted region involving the bilateral hippocampus. **B** Experimental outline. Transcranial LIPUS was applied 24 h after BCAS and subsequently daily with a stimulation time of 5 min at an ultrasound pressure of 0.51 MPa for a period of 28 days. **C** Fndc5 mRNA expression in hippocampus of ageing mice. **D** Irisin concentration in hippocampus of ageing mice. **E** Fndc5 mRNA expression in hippocampus of adult mice. **F** Irisin concentration in hippocampus of adult mice. **G** Representative Western blots of Fndc5/irisin in hippocampus of adult mice. **H** Hippocampal Fndc5/irisin levels normalized by β-actin in hippocampus of adult mice. **I** Fndc5 expression in cerebellum of ageing mice. **J** Fndc5 expression in cerebellum of adult mice. **K** Fndc5 expression in forebrain of ageing mice. **L** Irisin concentration in forebrain of ageing mice. **M** Fndc5 expression in forebrain of adult mice. Sham, sham-operated mice; BCAS, mice were subjected to BCAS injury; LIPUS, adult BCAS mice treated with transcranial low intensity pulsed ultrasound. (n = 8 per group. All data are expressed as mean ± SEM, *P < 0.05, **P < 0.01, ***P < 0.001, ns means no statistical significance)

Fndc5/irisin mediates the neuroprotective effects of exercise on brain [12]. LIPUS, as a new technology of neuromodulation, has the similar protective effects with the exercise on neuroinflammation and neurodegenerative diseases. Thus, we investigated the possible hypothesis that LIPUS may have the positive effect on modulating the activity of hippocampal Fndc5/irisin. LIPUS was performed according to an established regimen. Interestingly, LIPUS significantly increased the Fndc5 gene expression (*P* < 0.001; Fig. 3C) and irisin concentration (*P* = 0.004; Fig. 3D) in hippocampi of ageing BCAS mice. Consistently, the hippocampal Fndc5 gene expression (*P* < 0.001; Fig. 3E) and irisin concentration (*P* = 0.001; Fig. 3F) were enhanced by LIPUS in adult BCAS mice. Moreover, LIPUS significantly increased the protein expression of Fndc5/irisin in adult BCAS hippocampi (*P* = 0.001; Fig. 3G, H). The data of q-PCR, ELISA and Western blot indicated LIPUS drives a bolstering of hippocampal Fndc5/irisin level in mice with experimental VaD. Importantly, the worked range of LIPUS extended beyond the bilateral hippocampi in current study, so we further explored the effect of LIPUS on Fndc5/irisin in other regions, such as forebrain and cerebellum. Interestingly, LIPUS significantly increased the forebrain Fndc5 gene expression (*P* = 0.006; Fig. 3K) and irisin concentration (*P* = 0.049; Fig. 3L) in ageing BCAS mice, but not in adult mice with experimental VaD (Fndc5 mRNA of forebrain: *P* = 0.526; Fig. 3M, Fndc5 mRNA of cerebellum: *P* = 0.878; Fig. 3J). The activity of cerebellum Fndc5/irisin in both ageing and adult BCAS mice were not affected by LIPUS. Our finding indicated transcranial LIPUS seems to have a positive efficacy in restoring the VaD-induced reduction of Fndc5/irisin within its ultrasound working range.

### Fndc5/irisin mediates the neurorestorative effects of LIPUS on hippocampal damage and cognitive deficits in experimental VaD

**Fig. 4:**
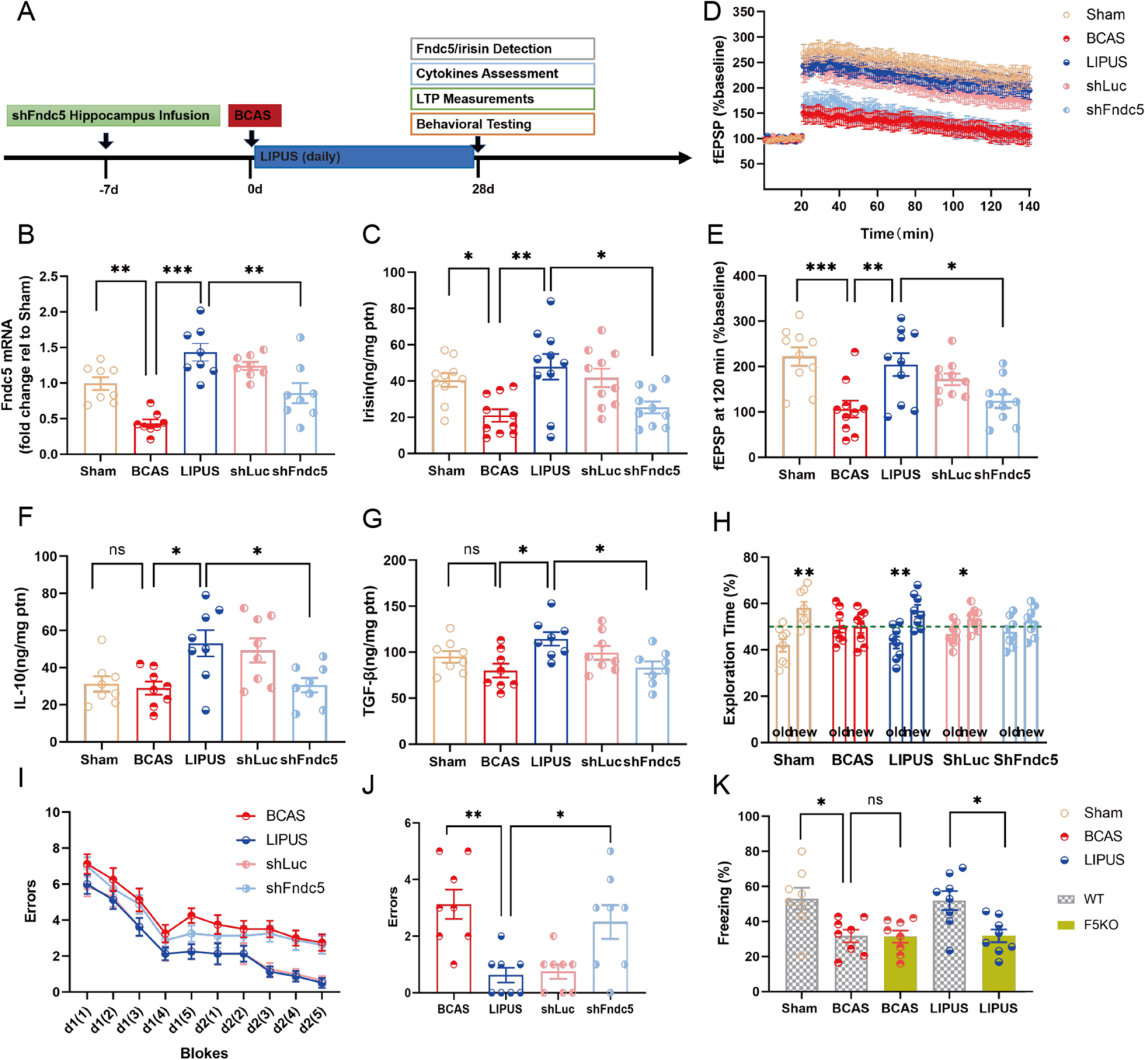
Fndc5/irisin mediates the neurorestorative effects of LIPUS on experimental VaD. **A** Experimental outline. Knockdown the expression of hippocampal Fndc5 using shRNA before 7days of BCAS. Transcranial LIPUS was applied 24 h after BCAS and subsequently daily with a stimulation time of 5 min at an ultrasound pressure of 0.51 MPa for a period of 28 days. **B**, **C** Fndc5 mRNA expression and irisin concentration in hippocampus of adult mice were respectively detected using qPCR and ELISA on day 28 after BCAS. **D** Hippocampal synaptic plasticity was analyzed using Patch Clamp, average traces of fEPSP in hippocampal slices collected on day 28 after BCAS. **E** The data of fEPSP at 120 min. **F**, **G** Hippocampal TGF-β and IL-10 were determined using ELISA on day 28 after BCAS. **H** Exploratory time on old or new object in novel object recognition test were illustrated in histogram. **I** The errors in radial arm water maze test were continuously measured in each block on day 28 after BCAS. **J** The data of errors in last block were counted in a histogram. **K** Global Fndc5 knock-out mice (F5KO) was employed to mechanism exploration, contextual fear test was used to assess cognitive deficit on day 28 after BCAS. the percentage of freezing time obtained on day 28 after BCAS were illustrated in histogram. Sham, sham-operated mice; BCAS, mice were subjected to BCAS injury; LIPUS, BCAS adult mice with LIPUS treatment; shFndc5 or shLuc, LIPUS-treated adult BCAS mice with intrahippocampal injection with lentiviral particles expressing shRNA against murine Fndc5 or luciferase. (n = 8 per group. All data are expressed as mean ± SEM, *P < 0.05, **P < 0.01, ***P < 0.001, ns means no statistical significance)

LIPUS is a novel neuromodulation technology and has gained increasing attention in the field of neuroscience over the past decades. Here, we investigated the therapeutic potential of LIPUS in VaD. In the patch clamp test, LIPUS rescued the damaged hippocampal synaptic plasticity by improving the fEPSPs of Ex vivo hippocampal slices (BCAS vs. LIPUS: *P* = 0.006; Fig. 4D, E). The production of TGF-β (BCAS vs. LIPUS: *P* = 0.011; Fig. 4G) and IL-10 (BCAS vs. LIPUS: *P* = 0.019; Fig. 4F), two anti-inflammatory cytokines, were significantly increased by LIPUS in hippocampus of BCAS mice. Expectantly, LIPUS-treated mice performed better in multiple neurobehavioral tests. In novel object recognition test, LIPUS improved the interest of BCAS mice on the new subject and increased the time consumption to explore the new subject (BCAS: *P* = 0.947, LIPUS: *P* = 0.002; Fig. 4H). Moreover, spatial learning and memory function of BCAS mice were continuously detected in ten blocks (five blocks per day) during two-day radial arm water maze test, the data of last block on day 2 indicated that LIPUS significantly decreased the numbers of entries into incorrect maze arms (errors) in BCAS mice (BCAS vs. LIPUS: *P* = 0.002; Fig. 4I, J). Collectively, LIPUS improved the hippocampal damage and cognitive deficits in BCAS mice.

To explored whether hippocampal Fndc5/irisin mediates the effects of LIPUS on VaD, bilateral intrahippocampal injection of shFndc5 was used to interfere the level of hippocampal Fndc5/irisin. Using ELISA and qPCR, shFndc5 infection successfully interfere the LIPUS-drove enhancement of hippocampal Fndc5/irisin in VaD mice (LIPUS vs. shFndc5: Fndc5 mRNA: *P* = 0.002; Fig. 4B; irisin concentration: *P* = 0.013; Fig. 4C), but shLuc (control) infection did not exhibit this effect (LIPUS vs. shLuc: Fndc5 mRNA: *P* = 0.649; Fig. 4B; irisin concentration: *P* = 0.886; Fig. 4C). Correspondingly, shFndc5 infection totally reversed the protective effects of LIPUS on hippocampal damage of VaD mice including the hippocampal synaptic plasticity (LIPUS vs. shFndc5: *P* = 0.034; Fig. 4D, E), and the hippocampal level of IL-10 (LIPUS vs. shFndc5: *P* = 0.032; Fig. 4F) and TGF-β (LIPUS vs. shFndc5: *P* = 0.026; Fig. 4G). Consistently, LIPUS-induced improvements on cognitive deficits of BCAS mice were significantly attenuated by shFndc5 infection. Compared to LIPUS group, the mice in shFndc5 group exhibited neglect of new subject in novel object recognition test (LIPUS: *P* = 0.002, shFndc5: *P* = 0.183; Fig. 4H), and had increased errors in radial arm water maze test (LIPUS vs. shFndc5: *P* = 0.024, Fig. 4I, J). However, the protective effects of LIPUS on BCAS mice were not reversed by the shLuc infection. The data of genetic knockdown of hippocampal Fndc5/irisin in vivo indicated Fndc5/irisin may mediate the protective effects of LIPUS on experimental VaD. We further verify this mechanism using a global Fndc5 knock-out mice (F5KO). In contextual fear test, global F5KO did not aggravate the cognitive deficits of BCAS mice, but significantly reversed the LIPUS-induced improvements on cognitive deficits of BCAS mice (*P* = 0.030; Fig. 4K). The data of F5KO mice further demonstrated Fndc5 mediates the protective effects of LIPUS on VaD.

### LIPUS increases spine density as well as irisin secretion via enhancing the Fndc5 expression in OGD hippocampal neurons

**Fig. 5:**
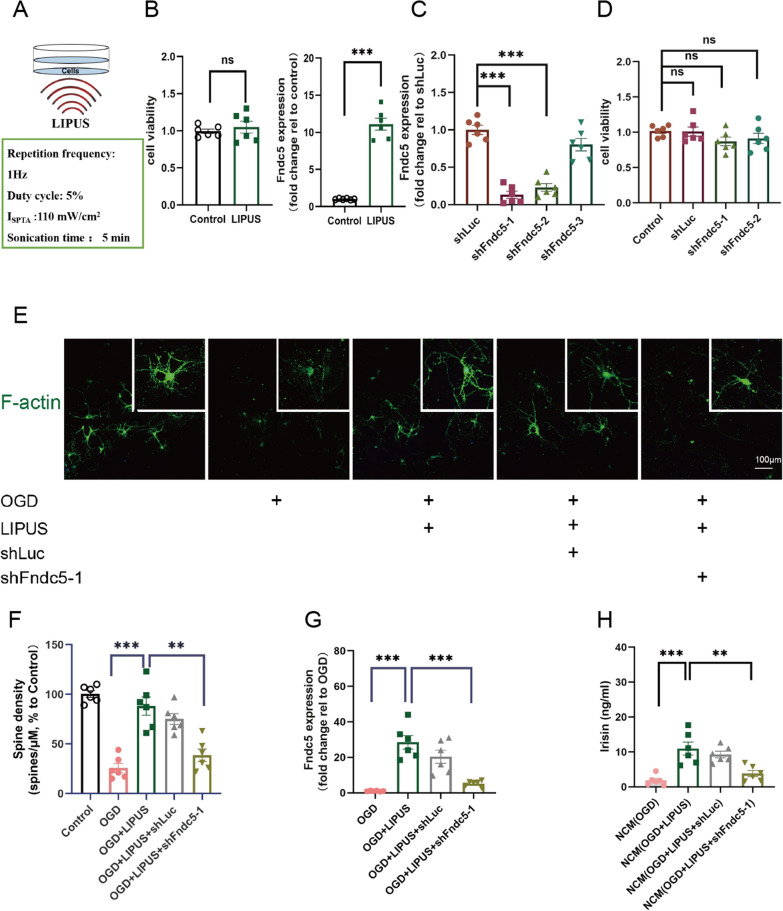
LIPUS increases spine density and irisin secretion via upregulating the Fndc5 level in hippocampal neurons. Primary hippocampal neurons were isolated from C57/BL6 wild-type E17 embryos. **A** Schematic diagram of LIPUS in vitro. LIPUS was daily applied for a stimulation time of 5 min with a I_SPTA_ of 110 mW/cm^2^ at a period of 2 days. **B** Primary hippocampal neurons at DIV 7 were treated with LIPUS and then harvested 2 days later. Cell viability of hippocampal neurons were assessed using CCK-8 Kit. Fndc5 expression of hippocampal neurons were assessed by qPCR. **C**, **D** Primary hippocampal neurons at DIV 5 were transduced with lentivirus carrying three specified shRNA hairpin against Fndc5 (shFndc5-1, shFndc5-2 and shFndc5-3) or luciferase (shLuc), then harvested 4 days later. Fndc5 expression was assessed by qPCR (**C**), Cell viability was assessed using CCK-8 Kit (**D**). **E**–**H** Primary hippocampal neurons at DIV 7 were injured by 6 h OGD, and then harvested 2 days later. Dendritic spines of primary hippocampal neurons were labeled using Phalloidin in Immunohistochemistry (**E**). Spine density of primary hippocampal neurons were analyzed between neurons with no treatment (control), neurons with OGD injure (OGD), OGD-injured neurons with LIPUS treatment (OGD + LIPUS), OGD-injured neurons with LIPUS treatment and shFndc5-1 infection (OGD + LIPUS + shFndc5-1), OGD-injured neurons with LIPUS treatment and shLuC infection (OGD + LIPUS + shLuc) (**F**). Fndc5 mRNA expression of hippocampal neurons with different interventions were analyzed in histogram (**G**). Irisin level in supernatants of hippocampal neurons with different interventions were detected using EILSA (**H**). Culture supernatants of hippocampal neurons with different interventions were further used as neuron-derived conditioned medium (NCM). (n = 6 per group, 6 independent experiments from 6 different neurons preps. All data are expressed as mean ± SEM, **P < 0.01, ***P < 0.001, ns means no statistical significance)

In addition to Ex vivo hippocampal slices, hippocampal neurons were employed to further assess the synaptic plasticity from a morphological perspective. LIPUS was performed (5 min, daily) at DIV 7 for 2 days, this scheme had no damage to hippocampal neurons (*P* = 0.528; Fig. 5B). Fndc5 can express in hippocampal neurons and has been shown to mediate endurance exercise-related neuroprotection. LIPUS increased the Fndc5 expression in hippocampal neurons (*P* < 0.0001; Fig. 5B). To investigate if Fndc5 is required for the effect of LIPUS on synaptic plasticity, lentivirally delivered shRNA was employed to interfere the Fndc5 expression in primary hippocampal neurons. To avoid possible off-targets of a single hairpin, we examined a total of three hairpins, two of which significantly reduced Fndc5 gene expression (shLuc vs. shFndc5-1: *P* < 0.0001, shLuc vs. shFndc5-2: *P* < 0.0001; Fig. 5C) and had no obviously damage to hippocampal neurons (control vs. shLuc, *P* > 0.05, control vs. shFndc5-1, *P* > 0.05, control vs. shFndc5-2, *P* > 0.05; Fig. 5D). OGD was used to mimic VaD ischemic damage, and induced a significant dendritic spine loss in hippocampal neurons. Consistent with our result of Ex vivo hippocampal slices, LIPUS significantly increased the spine density (OGD vs. OGD + LIPUS: *P* < 0.0001; Fig. 5F) and Fndc5 expression (OGD vs. OGD + LIPUS: *P* < 0.0001; Fig. 5G) in OGD-injured hippocampal neurons, while loss of function of Fndc5 via shRNA-mediated knockdown against Fndc5 reversed the protective effect of LIPUS on spine density in OGD-injured hippocampal neurons (OGD + LIPUS vs. OGD + LIPUS + shFndc5-1: *P* < 0.0001; Fig. 5F), suggesting LIPUS rescues dendritic spine loss may via enhancing the expression of Fndc5 in ischemic hippocampal neurons. Moreover, LIPUS-induced high expression of Fndc5 in hippocampal neurons arouses our interest to test whether Fndc5 is cleaved and secreted as irisin in hippocampal neurons. Interestingly, irisin was detected in supernatant of OGD-injured hippocampal neurons although the concentration was low. We further found the change of irisin level in supernatant correlated with the variety of Fndc5 expression in neurons (Fig. 5H), indicating LIPUS facilitates neuron-secreted irisin via increasing Fndc5 expression in ischemic hippocampal neurons. The supernatants of those neuron cultures were further used as neuron-derived conditioned medium (NCM).

### LIPUS raises the proportion of neuroprotective astrocytes in OGD hippocampal astrocyte cultures with the help of neuron-secreted irisin

**Fig. 6:**
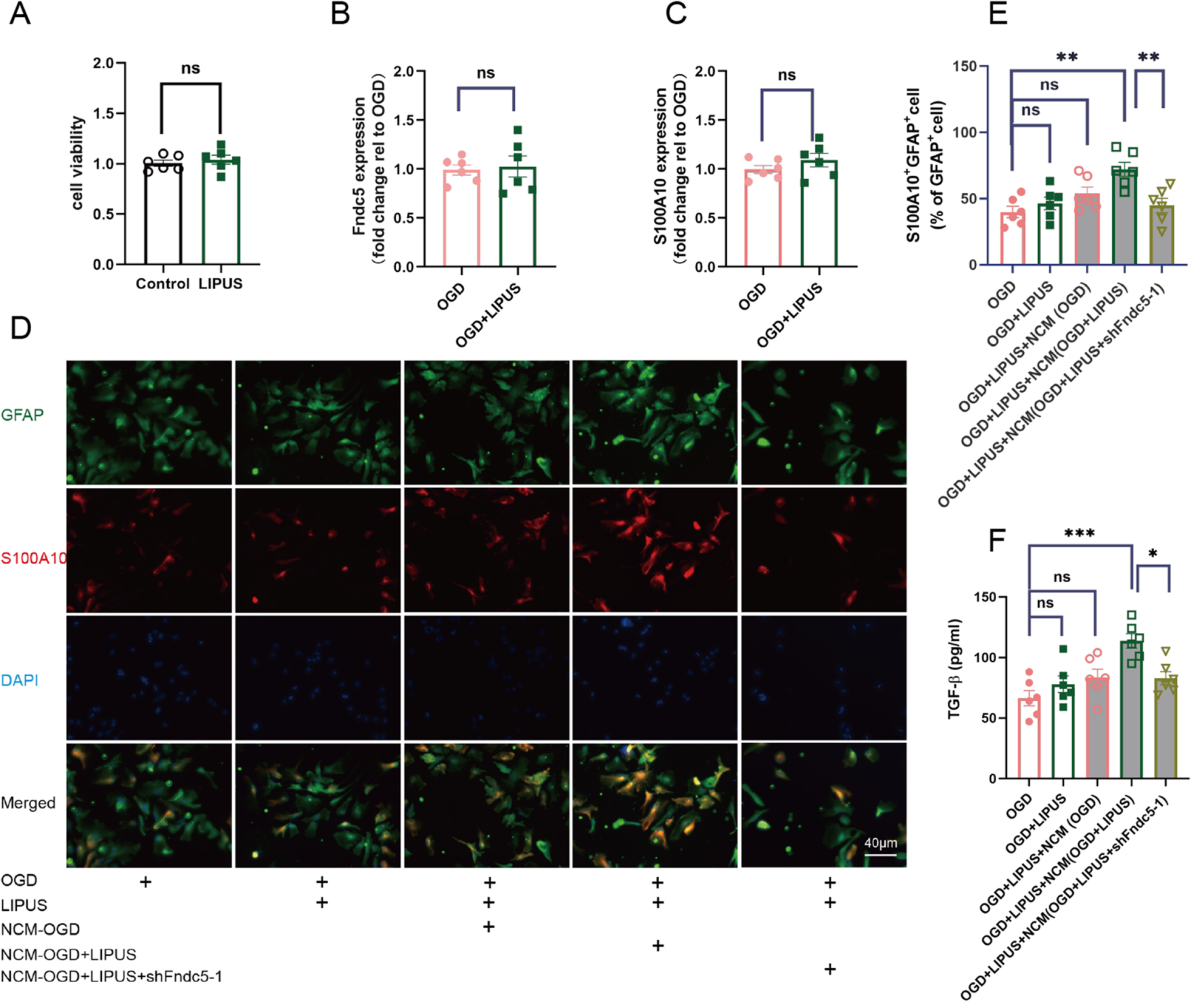
LIPUS enhances the proportion of neuroprotective astrocytes with the help of neuron-secreted Irisin. Primary hippocampal astrocytes were harvested from C57BL/6J mouse pups within 24 h after birth. **A** Primary hippocampal astrocytes at DIV 12 were treated with LIPUS and then harvested 2 days later. Cell viability was assessed using CCK-8 Kit. **B**, **C** Primary hippocampal astrocytes at DIV 12 were injured by OGD for 6 h and then harvested 2 days later. Fndc5 expression and S100A10 (neuroprotective phenotype marker) level were assessed by qPCR between the astrocytes with OGD injure and the astrocytes with OGD injure as well as LIPUS treatment. **D**– **F** Primary hippocampal astrocytes at DIV 12 were replaced medium with the NCM, simultaneously processed with 6 h OGD as well as 2 day LIPUS, and then then harvested 2days later. S100A10 and GFAP (reactive astrocyte marker) were used to determine neuroprotective astrocyte (S100A10 and GFAP co-labeled cell), the representative images of astrocytes in immunohistochemistry (**D**). The proportion of neuroprotective astrocytes were detected using the ImageJ software (**E**). The level of TGF-β in the supernatants of astrocytes were determined using ELISA (**F**). (n = 6 per group, 6 independent experiments from 6 different astrocytes preps. All data are expressed as mean ± SEM, *P < 0.05, **P < 0.01, ***P < 0.001, ns means no statistical significance)

Astrocytes are known to be involved in neuroinflammation [40] and may serve as potential targets for LIPUS [19]. Ischemic injury can induce the neuroprotective phenotype of reactive astrocytes [41], which ameliorates neuroinflammation by secreting anti-inflammatory cytokines, such as TGF-β [42, 43]. Therefore, hippocampal astrocytes were prepared to explore the effect of LIPUS on the neuroprotective phenotype of astrocytes and the related mechanisms. LIPUS was performed according to the same scheme of hippocampal neurons in hippocampal astrocyte cultures, and had no damage to hippocampal astrocytes (Control vs. LIPUS: *P* = 0.530; Fig. 6A). OGD injure was also used to mimic VaD ischemic damage in hippocampal astrocyte cultures. A most used neuroprotective marker (S100A10), and the reactive astrocytes marker (GFAP) were used to determine neuroprotective astrocytes [43]. Different from neurons, LIPUS did not increase the expressions of Fndc5 (OGD vs. OGD + LIPUS: *P* = 0.765; Fig. 6B) and S100A10 (OGD vs. OGD + LIPUS: *P* = 0.261; Fig. 6C) in OGD-injured hippocampal astrocytes, suggesting Fndc5 may not be the target mechanism of LIPUS in astrocytes. It is well known that irisin has excellent efficiency in controlling neuroinflammation [14]. Thus, we further guessed that LIPUS may facilitate neuroprotective phenotype of OGD-injured hippocampal astrocytes with the help of neuron-secreted irisin, and turned to replacement the astrocyte medium with NCM. Interestingly, LIPUS successfully increased the proportion of S100A10 and GFAP co-labeled cells in OGD-injured hippocampal astrocytes cultured in NCM(OGD + LIPUS) with the high level of neuron-secreted irisin (OGD vs. OGD + LIPUS + NCM(OGD + LIPUS): *P* = 0.001; Fig. 6D, E), while failed to enhance the proportion of neuroprotective astrocytes in other NCM (OGD vs. OGD + LIPUS + NCM(OGD): *P* = 0.313; Fig. 6D, E) or astrocyte medium (OGD vs. OGD + LIPUS: *P* = 0.886, Fig. 6D, E). Consistent with the result of immunohistochemistry, LIPUS combined with neuron-secreted irisin from NCM(OGD + LIPUS) significantly raised astrocyte-secreted TGF-β in OGD-injured hippocampal astrocytes (OGD vs. OGD + LIPUS + NCM(OGD + LIPUS): *P* < 0.001; Fig. 6F). The above data suggested that neuron-secreted irisin may be involved in the effect of LIPUS on facilitating the switch of hippocampal astrocytes towards neuroprotective phenotype.

### Irisin drives OGD astrocytes toward neuroprotective phenotype

**Fig. 7:**
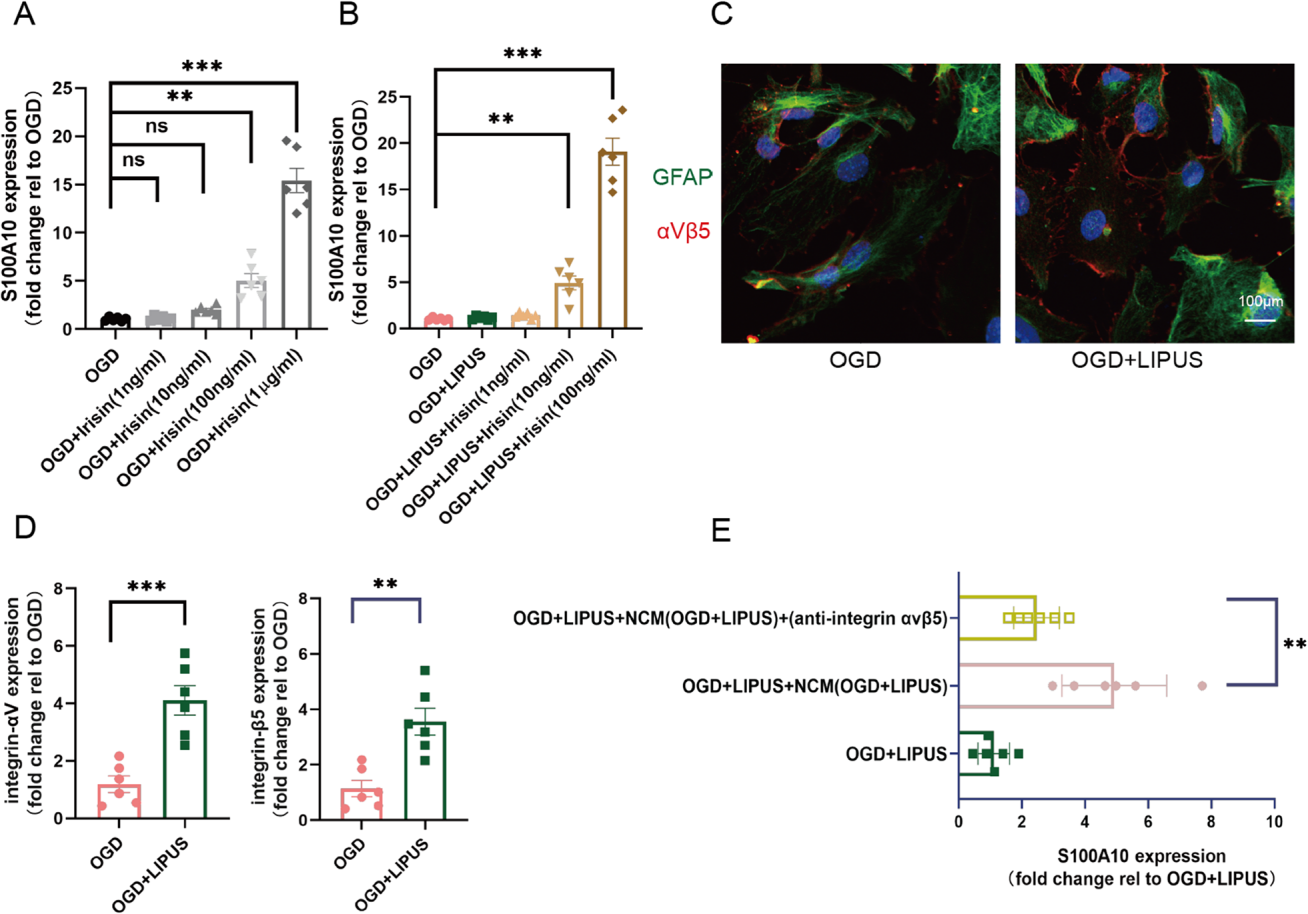
Irisin drives reactive astrocytes toward neuroprotective phenotype. **A** Primary hippocampal astrocytes at DIV 12 were injured by OGD for 6 h, treated by recombinant irisin with different concentration, and then harvested 2days later. S100A10 level were assessed by qPCR. **B** Primary hippocampal astrocytes at DIV 12 were injured by OGD for 6 h, treated with LIPUS or/and recombinant irisin with different concentration, then harvested 2days later. S100A10 level were assessed by qPCR. **C** Representative images of primary astrocytes (green) expressing integrin-αVβ5 (red) in immunohistochemistry. **D** The integrin-αV and integrin-β5 level were determined using qPCR between the astrocytes with OGD injure and the astrocytes with OGD injure as well as LIPUS treatment. **E** Primary hippocampal astrocytes at DIV 12 were replaced medium with the NCM (OGD + LIPUS), simultaneously processed with 6 h OGD as well as 2 day LIPUS or/and the anti-integrin-blocking antibodies. S100A10 mRNA level were assessed by qPCR. (n = 6 per group, 6 independent experiments from 6 different astrocytes preps. All data are expressed as mean ± SEM **P < 0.01, ***P < 0.001, ns means no statistical significance)

To further investigate the efficacy of irisin in modulating neuroprotective astrocytes, OGD-injured hippocampal astrocytes were incubated with recombinant irisin at different concentrations. We found irisin can increase the S100A10 expression of OGD-injured hippocampal astrocytes in a concentration-dependent manner, only irisin at high concentrations (100nM or 1µM) take effect (OGD vs. OGD + Irisin(100ng/ml): *P* = 0.002, OGD vs. OGD + Irisin(1 µg/ml): *P* < 0.001; Fig. 7A). Interestingly, when LIPUS and recombinant irisin treating together, irisin at 10 ng/ml successfully increased the S100A10 expression of OGD-injured hippocampal astrocytes (OGD vs. OGD + LIPUS + Irisin(10ng/ml): *P* = 0.007; Fig. 7B), suggesting LIPUS may improve the efficiency of irisin in driving neuroprotective astrocytes. It has been indicated that integrin-αVβ5 can express in activated astrocytes [29] and as be a possible cellular receptor of irisin [44]. Using immunohistochemistry, integrin-αVβ5 indeed expressed in OGD-injured hippocampal astrocytes, and seemed to express more in LIPUS-treated OGD astrocytes (Fig. 7C). Moreover, we found LIPUS enhances the mRNA expression of integrin-αV and integrin-β5 in OGD-injured hippocampal astrocytes (OGD vs. OGD + LIPUS: *P* < 0.001 and *P* = 0.002; Fig. 7D). Interestingly when a neutralizing antibody(10 µg/ml) for integrin αVβ5 (Anti-Integrin αVβ5 antibody, Abcam, Cambridge, UK) was added in NCM (OGD + LIPUS), the promoted effect of neuron-secreted irisin in LIPUS-mediated increase of the S100A10 expression in OGD-injured hippocampal astrocytes was almost reversed (*P* = 0.004; Fig. 7E). These data indicated irisin drives OGD-injured hippocampal astrocytes to neuroprotective phenotype may via LIPUS-induced high expression of the integrinα-αVβ5.

## Discussion

Nowadays, population aging is an unavoidable problem, and the elderly are going through dementia, such as AD and VaD [45]. Physical exercise has been demonstrated to induce memory-related events in the brain, and proposed as a positive approach to protect against dementia from early stage to late stage, potentially bringing about remarkable benefits for dementia prevention and treatment [46]. Fndc5/irisin signaling has been identified as the endogenous molecule mechanism responsible for the beneficial effects of exercise on cognition [13, 29]. Recent discoveries have suggested potential roles of Fndc5/irisin in the cognitive impairments, with effects potentially related to the AD [8]. Lourenco et al. elucidated that brain Fndc5/irisin is decreased in AD patients and experimental models, while there is no significant decline of irisin in serum [15]. In present study, we offered the first evidence on the state of Fndc5/irisin in the BCAS mice, which was similar to data showed by Lourenco et al. in the AD models. The level of Fndc5/irisin was reduced in CSF, forebrain, hippocampus and cerebellum of ageing BCAS mice, while only in hippocampus of the adult BCAS mice. These data suggested CCH destroys the activity of Fndc5/irisin in brain, especially in the hippocampal region, this pathological process may be accelerated or aggravated by age burden. Moreover, accumulating evidences had highlighted the positive strategy of rescuing or improving the signaling pathway activity of brain Fndc5/irisin in dementia prevention and treatment [39, 47]. Excitingly, we further demonstrated that boosting hippocampal Fndc5/irisin improves the synaptic plasticity of ischemic neurons and the inflammatory microenvironment in hippocampus, and then reduces the cognitive deficits in BCAS mice. Thus, it is convinced that hippocampal Fndc5/irisin plays crucial roles in the pathophysiology of BCAS model, indicating that Fndc5/irisin could represent an attractive novel strategy aimed to treat VaD patients, including those who are unable or unfit to engage in regular physical exercise. Many patients in late stage of VaD are disabled owing to other age-related conditions or the disease-related complications (e.g., limb dysfunction, visual problems, depression) that preclude them from obtaining the benefits of exercise. Therefore, developing alternative approaches based on the neuroprotective effects of exercise on the brain might be able to benefit those patients.

There are still not many effective medications for brain diseases, which is partially due to problems for novel drugs failing to cross the blood-brain barrier (BBB) [48]. Application of surgery is limited because of invasiveness, particularly in the elderly. Brain stimulation is considered as a third therapeutic method for support of restorative brain processes [49]. However, available electrophysiological techniques are sometimes limited regarding targeting and accessing to the deep brain regions. Improvement for the above three approaches is now possible by a novel development: transcranial ultrasound [17]. Recently, technologies regarding transcranial ultrasound have been obviously improved and a novel pulsed stimulation strategy has been introduced, which can guarantee the safe of transcranial ultrasound application [17]. High intensity transcranial focused ultrasound is used to destruct pathologically brain tissue [50]. Transcranial LIPUS has been confirmed to induce a focal and reversible opening of BBB, driving a clearance of pathological proteins in brain or allowing a brain targeted delivery of drug or gene therapy [51]. Another therapeutic option of transcranial LIPUS concerns non-invasive and targeted neuromodulation without affecting BBB, which allows improvement of impaired brain functions [17, 52]. In present study, we focused on the neurorestorative effect of transcranial LIPUS (parameter tuning to avoid opening BBB). Expectedly, LIPUS exists an exact efficacy to rescue the reduced Fndc5/irisin in both adults and ageing BCAS brains, and enhance the Fndc5 expression in OGD-injured hippocampal neurons. However, both adenovirus intracerebroventricular injection and recombinant irisin bilateral intrahippocampal injection are not clinically acceptable strategy for boosting brain Fndc5/irisin, thus LIPUS might be a valuable alternative approach to improve the impaired state of brain Fndc5/irisin in the CCH injure. Simultaneously, we reported previously unstated properties of LIPUS in improvement of impaired neuron synaptic plasticity and inflammatory microenvironment in hippocampus injured by CCH, which were accompanied by the enhancement of Fndc5/irisin in ischemic hippocampus. Mechanistically, using genetic interference or knockout techniques targeting Fndc5, we revealed hippocampal Fndc5/irisin may mediates the beneficial effects of LIPUS on hippocampal damage and cognitive deficits in experimental VaD. Additionally, we offered explanation for preliminary mechanisms regarding the role of Fndc5/irisin in the LIPUS-induced protection against VaD using hippocampal primary cell cultures: LIPUS promotes hippocampal neurons to secrete irisin by increasing Fndc5 expression, those neuron-secreted irisin directly accelerate synaptic plasticity, and help LIPUS to drive astrocytes toward neuroprotective phenotype, then alleviating cognitive impairment. These data suggested transcranial LIPUS exists a promising therapeutic potential for VaD may through the neurorestorative effect and the anti-inflammatory property of Fndc5/irisin signaling in hippocampus. It is worth noting that the upstream or downstream mechanisms regarding the Fndc5/irisin by which transcranial LIPUS induce such neurorestoration in BCAS mice are largely unclear. Furthermore, both Fndc5-dependent and Fndc5-independent signaling pathways by which transcranial LIPUS induces protection of brain damage associated with CCH seem plausible. Thus, future study is needed to elucidate the detailed molecules and pathways involved in transcranial LIPUS-induced neuroprotection in BCAS mice.

Irisin is now considered as a newly exercise-related hormone mainly secreted from muscle and a tiny portion of adipose tissue [9, 53]. The existence of irisin in various brain regions and cellular groups [54] raises an interesting question about the irisin secretion in inherent brain cells, such as neurons and astrocytes. In 2013, hippocampal neurons of exercise mice were reported expressed Fndc5, which was known as the parent protein of irisin [13]. In present study, we provided the clear evidence that irisin can secrete from the hippocampal neurons, albeit at low concentrations. LIPUS enhanced the Fndc5 expression in OGD-injured hippocampal neurons as well as the concentration of irisin in the supernatants of OGD-injured hippocampal neuron cultures, these effects of LIPUS were reversed by the shRNA targeting Fndc5. Our data suggested LIPUS promoted the secretion of neuron-derived irisin may via regulating the expression of its parent protein Fndc5. The systemic use of exogenous irisin is impeded by its potential unknown risks. Thus, the implementation of potential strategies for boosting the endogenous irisin in a specific brain region would be highly desirable.

Recently, the anti-inflammatory property of irisin has received continuous attention in neuroscience [14]. Clear evidence reported that irisin exerted its anti-inflammatory effects via downregulating NF-κB pathway, decreasing multiple proinflammatory cytokines in adipocyte 3T3 L1 cell line, thus alleviating the obesity-related neuroinflammation [55]. Additionally, irisin was proved to improve memory in diabetic mice by declining the level of IL-1β and IL-6 in the murine hippocampus, the underlying mechanism in which was by downregulating the STAT3 and NF-κB pathways [56]. Furthermore, irisin has been suggested to have a positive efficacy in the phenotypic switch of macrophages in adipose tissue to modulate neuroinflammation [57]. Importantly, Wang Yao et al. reported irisin regulates the phenotypic switch of brain microglia/macrophage from pro-inflammatory polarization to anti-inflammatory polarization through the integrin αVβ5/AMPK signaling pathway in intracerebral hemorrhage models [44]. In this study, bilateral intrahippocampal injection of recombinant irisin improved hippocampal inflammatory microenvironment of BCAS mice by increasing the level of anti-inflammatory cytokines, and direct adding recombinant irisin in OGD-injured hippocampal astrocyte cultures successfully enhance the proportion of neuroprotective astrocytes. Our data revealed the possible previously unknown properties of irisin in regulating the phenotypic switch of astrocytes, which may be a novel effective therapeutic strategy against neuroinflammation. The integrin-αVβ5 also seems to be related to the underlying mechanism by which irisin regulates the astrocyte phenotype, this preliminary mechanism exploration might provide valuable hints for future study focusing on the astrocyte-mediated neuroinflammation.

Our study revealed an interesting relation between the LIPUS and the Fndc5/irisin signaling in hippocampus under the pathology caused by CCH, but we are currently unable to provide more direct evidence due to some related experimental technical limitations. Transcranial focused LIPUS can target deep brain region such as hippocampus in rats and humans [58, 59], but is difficult to implement in mice. Moreover, genetic approach for tissue-specific or cell-specific knockout of Fndc5 is still lacking [9]. Further studies using the transcranial focused LIPUS with precise targeting mouse hippocampus and the hippocampus-specific knockout of Fndc5 are need and help to further confirm the beneficial efficacy of LIPUS in hippocampal Fndc5/irisin signaling.

## Conclusion

Fndc5/irisin is reduced in hippocampus of dementia mice with CCH. LIPUS exists a positive efficacy in enhancing the hippocampal Fndc5/irisin in experimental VaD. Hippocampal Fndc5/irisin signaling mediates the neurorestorative effect of LIPUS on VaD mice. Hippocampal Fndc5/irisin signaling might be a promising therapeutic target for treating the brain disorders associated with CCH. Moreover LIPUS-induced activation of hippocampal Fndc5/irisin signaling could be an alternative strategy to drive the benefits of exercise for dementia patients, especially the patients who are disabled.

## Data Availability

The datasets used and/or analysed in the current study are available from the corresponding authors on reasonable request.

## Abbreviations

AD: Alzheimer's disease
BBB: Blood-brain barrier
BCAS: Bilateral common carotid artery stenosis
BDNF: Brain-derived neurotrophic factor
CCH: Chronic cerebral hypoperfusion
CSF: Cerebrospinal fluid
fEPSPs: Field excitatory postsynaptic potentials
Fndc5: Fibronectin type III domain containing 5
GFAP: Glial fibrillary acidic protein
IL: Interleukin
LIPUS: Low-intensity pulsed ultrasound
NCM: Neuron-derived conditioned medium
OGD: Oxygen and glucose deprivation
S100A10: S100 calcium-binding protein A10
TGF: Transforming growth factor
VaD: Vascular dementia
